# Supplementary material for: Concomitant Retinal Alterations in Neuronal Activity and TNFα Pathway Are Detectable during the Pre-Symptomatic Stage in a Mouse Model of Alzheimer’s Disease
Source: Cells. 2022 May 16;11(10):1650. doi: 10.3390/cells11101650 (PMC9140134; doi:10.3390/cells11101650)
Supplement: Supplementary file 1 [file cells-11-01650-s001.zip › cells-1660765 SI.pdf]

Supplemental Figure S1. Schematic representation of retinal structure and electroretinogram (ERG) analysis. RPE: Retinal Pigment Epithelia; ONL: Outer Nuclear Layer; INL: Inner Nuclear Layer and GCL: Ganglion Cells Layer; PR: Photoreceptor cells.

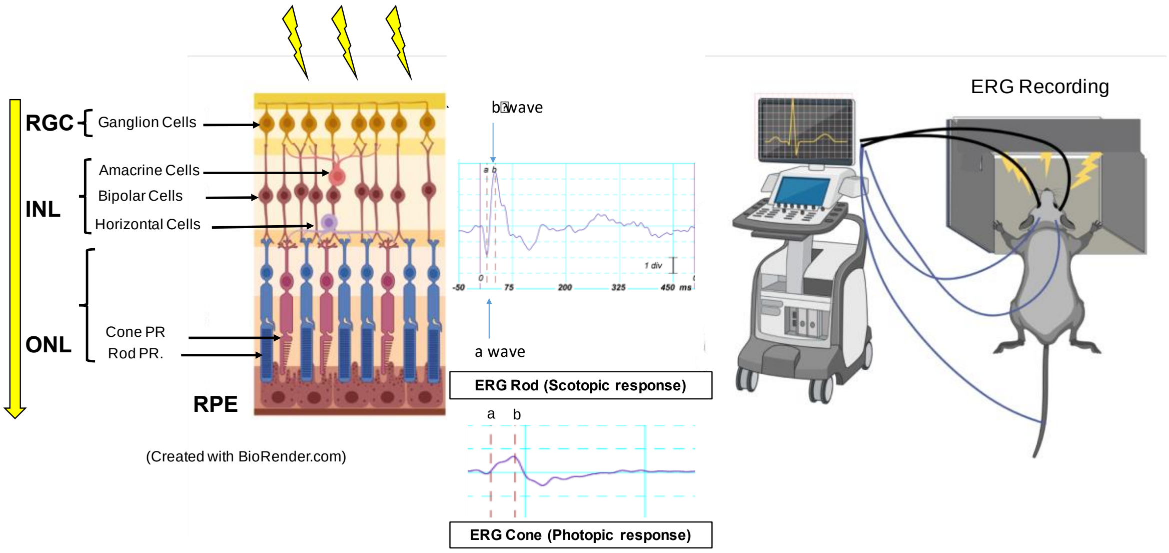

Supplemental Table S1. Number of mice for both genotypes with relevant gender data and statistics for comparison between sexes (biochemical and immunohistochemical experiments). Total number of mice used to generate the data shown in each figure is provided together with the number of female and male mice for both genotypes: wild-type (WT) and transgenic (TG) APP/PS1 mice. F=Female; M=male.

Fig.	WT	Mean+/- SEM		TG	Mean +/-SEM	
**1A′**	**N total=6**	**0.0003+/- 0.03**		**N total=5**	**0.26+0.08**	
	F=3	0.07+/- 0.005	**F vs M (p)**	F=2	0.24+/-0.06	**F vs M (p)**
	M=3	-0,07 +/-0 .006	0.1	M=3	0.27+/-0.15	0.99
**1A″**	**N total=6**	**0.0003+ 0.015**		**N total=4**	**1.07+/-0.19**	
	F=3	-0.008+/-0.03	**F vs M (p)**	F=1	1.51+/-0	**F vs M (p)**
	M=3	0.008+0.01	0.7	M=3	0.92+/-0.17	/
**1C (Ab42)**	**N total=3**	**0.71+/-0**		**N total=8**	**2.68+/-0.06**	
	F=1	0.63+/-0	**F vs M (p)**	F=2	2.76+/-0.13	**F vs M (p)**
	M=2	0.75+/- 0.08	/	M=6	2.66+/-0.08	0.4
**1C (Ab40)**	**N total=3**	**5.62+ 2.52**		**N total=8**	**55.85+/-1.83**	
	F=1	3.08+/-0	**F vs M (p)**	F=2	57.48+/-6.04	**F vs M (p)**
	M=2	6.47+/- 3.36	/	M=6	55.31+/-1.9	0.85
**1B′**	**N total=4**	**-0.0002+/- 0.02**		**N total=5**	**0.32+/-0.08**	
	F=1	0.063+/- 0	**F vs M (p)**	F=2	**0.15+/-0.02**	**F vs M (p)**

	M=3	-0.02 +/-0.003	/	M=3	**0.43+/-0.08**	**0.2**
**1B''**	**N total=5**	0+/- 0.04		**N total=5**	0.3+ 0.05	
	F=2	-0.05 +/-0.03	**F vs M (p)**	F=2	0.29 +0.15	**F vs M (p)**
	M=3	0.03 +/-0.07	0.5	M=3	0.31 +0.03	0.99
**1D (3-4M)**	**N total=4**	**0**		**N total=3**	**188.2+/-21.7**	
	F=2	0	**F vs M (p)**	F=1	192.9+/-0	**F vs M (p)**
	M=2	0	0.99	M=2	185.8+/-37.3	/
**1D (9-12M)**	**N total=3**	**18.33+/-2.3**		**N total=4**	**286.1+/-19.3**	
	F=1	19+/-0	**F vs M (p)**	F=2	269.2+/-38.48	**F vs M (p)**
	M=2	18+/-4	/	M=2	303+/-14	**0.66**
**3B Iba1**	**N total=6**	**1.00+/-0.26**		**N total=4**	**1.81+/- 0.26**	
	F=3	1.07+/-0.10	**F vs M (p)**	F=2	1.84+/-0.27	**F vs M (p)**
	M=3	0.93+/-0.2	0.99	M=2	1.78+/-0.15	0.99
**3B GFAP**	**N total=6**	**1.00+/-0.67**		**N total=4**	**4.28+ 1.27**	
	F=3	0.80+/-0.12	**F vs M (p)**	F=2	4.43+/-1.17	**F vs M (p)**
	M=3	1.19+/-0.56	0.99	M=2	4.13+/-1.00	0.66
**4A**	**N total=4**	**1.00+/-0.1**		**N total=6**	**1.09+/-0.17**	
	F=2	0.98+/-0.24	**F vs M (p)**	F=3	1.27+/-0.26	**F vs M (p)**
	M=2	1.02+/-0.04	0.99	M=3	0.92+/-0.22	0.4
**4B**	**N total=7**	**0.21+/-0.02**		**N total=6**	**0.33+/-0.03**	
	F=3	0.19+/-0.02	**F vs M (p)**	F=3	0.37+/-0.06	**F vs M (p)**
	M=4	0.21+/-0.03	**0.99**	M=3	0.29+/-0.03	**0.4**
**4C**	**N total=5**	**0.18+/-0.14**		**N total=5**	**7.48+2.9**	
	F=4	0.22+/-0.17	**F vs M (p)**	F=3	7.26+3.26	**F vs M (p)**
	M=1	0+/-0	/	M=2	7.8+ 7.5	0.99
**5A**	**N total=6**	**1.00+/-0.14**		**N total=9**	**2.27+/-0.26**	
	F=3	1.11+/-0.09	**F vs M (p)**	F=5	2.35+/-0.39	**F vs M (p)**
	M=3	0.89+/-0.28	0.99	M=4	2.17+/-0.38	0.55
**5B**	**N total=5**	**0.03+/-0.01**		**N total=7**	**0.10+/-0.01**	
	F=2	0.05+/-0.03	**F vs M (p)**	F=4	0.11+/-0.02	**F vs M (p)**
	M=3	0.02+/-0.005	0.4	M=3	0.08+/-0.02	0.22
**5D**	**N total=4**	**360.5+/-32.1**		**N total=3**	**639.3+/-54.4**	
	F=4	360.5+/-32.1	**F vs M (p)**	F=0		**F vs M (p)**
	M=0		/	M=3	639.3+/-54.4	/
**6A**	**N total=5**	**0.55+/-0.04**		**N total=9**	**0.53+/-0.02**	
	F=2	0.53+/-0.07	**F vs M (p)**	F=5	0.51+/-0.03	**F vs M (p)**
	M=3	0.55+/-0.05	0.99	M=4	0.56+/-0.03	0.25
**6B**	**N total=9**	**0.92+/-0.03**		**N total=9**	**0.90+/-0.03**	
	F=4	1.00+/-0.03	**F vs M (p)**	F=4	0.96+/-0.08	**F vs M (p)**

	M=5	0.85+/-0.04	0.06	M=5	0.85+/-0.01	0.28
**6C**	**N total=9**	**0.07+/-0.006**		**N total=9**	**0.23+/0.01**	
	F=4	0.06+/0.01	**F vs M (p)**	F=4	0.21+/0.01	**F vs M (p)**
	M=5	0.07+/0.007	0.44	M=5	0.26+/0.03	0.19
**6D**	**N total=9**	**0.07+ 0.006**		**N total=9**	**0.26+0.02**	
	F=4	0.06+ 0.008	**F vs M (p)**	F=4	0.22+ 0.03	**F vs M (p)**
	M=5	0.08+ 0.008	0.28	M=5	0.29+ 0.03	0.19

Supplemental Table S2. Number of mice for both genotypes with relevant gender data and statistics for data comparison between sexes (ERG experiments). Scotopic conditions: WT mice n=15 (8 F and 7 M) and TG: n=13 (7 F and 6 M). Photopic conditions: WT mice n=15 (8 F and 7 M) and TG: n=13 (7 F and 6 M). F=Female; M=male. p: *<0.05, **< 0.005, ***< 0.001, **** <0.0001.

Scotopic	WT	0.04 Cd.s/m^2	WT vs TG (p)	0.32 Cd.s/m^2	WT vs TG (p)	3.19 Cd.s/m^2	WT vs TG (p)	8 Cd.s/m^2	WT vs TG (p)
Fig.2B	**N total=15**	**2.556+/-0.6261**	**0.3373**	**16.81+/-1.732**	**0.0249***	**86.8+/-18.36**	**0.2650**	**115.5+/-15.04**	**0.5655**
	F=8	3+/-0.7	F vs M (P)	15.33+/-0.9	F vs M (P)	97.4+/-23	F vs M (P)	110+/-20.6	F vs M (P)
	M=7	2.2+/-0.5	0.2699	18.29+/-2.1	0.2686	76.22+/-11.6	0.9510	120.4+/-9.9	0.6458
Fig.2C	**N total=15**	**1.824+/-0.3692**	**<0.0001******	**18.3+/-1.956**	**0.7011**	**37.9+/-6.298**	**<0.0001*****	**45.3+/-5.033**	**<0.0001******
	F=8	1.571+/-0.6	F vs M (P)	17.4+/-1.5	F vs M (P)	35.83+/-6	F vs M (P)	45.5+/-6.2	F vs M (P)
	M=7	2.077+/-0.4	0.5282	19.3+/-2.7	0.7891	40.08+/-8.5	0.9322	45.17+/-6.131	0.7309
Fig.2D	**N total=15**	**48.7+/-6.636**	**0.0724**	**143+/-20.52**	**0.8019**	**272.6+/-50.81**	**0.8198**	**314.8+/-52.84**	**0.5358**
	F=8	46+/-3.3	F vs M (P)	144.7+/-20.8	F vs M (P)	268.4+/-51.03	F vs M (P)	329.9+/-53	F vs M (P)
	M=7	51.4+/-10.25	0.7733	141.2+/-18.9	0.6037	276.9+/-48.46	0.5054	299.6+/-67.04	0.8912
Fig.2E	**N total=15**	**25.6+/-4.85**	**<0.0001******	**89.9+/-13.73**	**0.0033****	**128.6+/-19.87**	**0.0013****	**153.2+/-21.38**	**0.0028****
	F=8	26.88+/-3.08	F vs M (P)	103.4+/-16.36	F vs M (P)	131+/-24.03	F vs M (P)	150.9+/-24.5	F vs M (P)
	M=7	24.33+/-6.8	0.0969	76.57+/-9	0.4811	126.3+/-14.52	0.8208	155.6+/-17.7	0.6832

Scotopic	TG	0.04 Cd.s/m²	WT vs TG (p)	0.32 Cd.s/m²	WT vs TG (p)	3.19 Cd.s/m²	WT vs TG (p)	8 Cd.s/m²	WT vs TG (p)
Fig. 2B	**N total=13**	**4.143+/- 0.919**	**0.3373**	**30.4+/- 4.052**	**0.0249***	**113.2+/- 19.61**	**0.2650**	**143.7+/- 21.43**	**0.5655**
	F=7	4.57+/- 1.251	F vs M (P)	31.8+/- 4.348	F vs M (P)	93.88+/- 17.35	F vs M (P)	127.1+/- 19.61	F vs M (P)
	M=6	3.714+/- 0.9689	0.5646	29+/- 1.279	0.4431	132.5+/- 20.88	0.0985	160.4+/- 33.16	0.5224
Fig. 2C	**N total=13**	**9.7+/- 1.238**	**<0.0001********	**22.3+/- 4.313**	**0.7011**	**88.44+/- 10.57**	**<0.0001*******	**132.7+/- 13.34**	**<0.0001********
	F=7	9.625+/- 0.92	F vs M (P)	20.67+/- 3.387	F vs M (P)	85.43+/- 10.31	F vs M (P)	131.2+/- 10.46	F vs M (P)
	M=6	9.929+/- 1.238	0.9794	24+/- 4.313	0.5509	91.46+/- 10.57	0.7456	134.2+/- 13.34	0.8715
Fig. 2D	**N total=13**	**80.1+/- 10.53**	**0.0724**	**205.8+/- 42.34**	**0.8019**	**266.95+/- 51.57**	**0.8198**	**295.5+/- 50.98**	**0.5358**
	F=7	77.27+/- 12.75	F vs M (P)	216+/- 39.23	F vs M (P)	271.7+/- 57.8	F vs M (P)	305.4+/- 53.33	F vs M (P)
	M=6	83+/- 14.81	0.8768	195.7+/- 51.66	0.5168	262.2+/- 66.02	0.7013	285.7+/- 49.25	0.5551
Fig. 2E	**N total=13**	**73.9+/- 10.57**	**<0.0001********	**203+/- 32.09**	**0.0033****	**302.45+/- 42.08**	**0.0013****	**319.8+/- 43.38**	**0.0028****
	F=7	74.32+/- 9.61	F vs M (P)	200.2+/- 25.98	F vs M (P)	296.3+/- 40.21	F vs M (P)	332.6+/- 42.48	F vs M (P)
	M=6	73.67+/- 11.19	0.9120	205.8+/- 41.34	0.9004	308.6+/- 44.06	0.8364	307.0+/- 39.24	0.6820

Photopic	WT	8 Cd.s/m²		TG	8 Cd.s/m²		WT vs TG (p)
**Fig.2G (3-4M)**	**N total=15**	**51.8+/-10.14**		**N total=13**	**55.14+/-7.622**		**0.8493**
	F=8	56.75+/-5.164	F vs M (P)	F=7	51.78+/-10.49	F vs M (P)	
	M=7	47+/-7.161	0.1849	M=6	58.5+/-11.72	0.6889	
**Fig.2G (9-12M)**	**N total=15**	**29.07+/-5.685**		**N total=13**	**44.3+/-5.606**		**0.0478***
	F=8	28.14+/-8.44	F vs M (P)	F=7	45.29+/-9.403	F vs M (P)	
	M=7	30+/-8.238	0.5082	M=6	43.38+/-7.161	0.9551	
